# Differential Signaling and Sugar Exchanges in Response to Avirulent Pathogen- and Symbiont-Derived Molecules in Tobacco Cells

**DOI:** 10.3389/fmicb.2017.02228

**Published:** 2017-11-20

**Authors:** Carole Pfister, Stéphane Bourque, Odile Chatagnier, Annick Chiltz, Jérôme Fromentin, Diederik Van Tuinen, Daniel Wipf, Nathalie Leborgne-Castel

**Affiliations:** Agroécologie, AgroSup Dijon, CNRS, INRA, Université Bourgogne Franche-Comté, Dijon, France

**Keywords:** chitotetrasaccharide, cryptogein, plant–microbe interactions, calcium, ROS, MAPKs, sugar transport, *Nicotiana tabacum*

## Abstract

Plants interact with microbes whose ultimate aim is to exploit plant carbohydrates for their reproduction. Plant–microbe interactions (PMIs) are classified according to the nature of their trophic exchanges: while mutualistic microbes trade nutrients with plants, pathogens unilaterally divert carbohydrates. The early responses following microbe recognition and the subsequent control of plant sugar distribution are still poorly understood. To further decipher PMI functionality, we used tobacco cells treated with microbial molecules mimicking pathogenic or mutualistic PMIs, namely cryptogein, a defense elicitor, and chitotetrasaccharide (CO4), which is secreted by mycorrhizal fungi. CO4 was perceived by tobacco cells and triggered widespread transient signaling components such as a sharp cytosolic Ca^2+^ elevation, NtrbohD-dependent H_2_O_2_ production, and MAP kinase activation. These CO4-induced events differed from those induced by cryptogein, i.e., sustained events leading to cell death. Furthermore, cryptogein treatment inhibited glucose and sucrose uptake but not fructose uptake, and promoted the expression of *NtSUT* and *NtSWEET* sugar transporters, whereas CO4 had no effect on sugar uptake and only a slight effect on *NtSWEET2B* expression. Our results suggest that microbial molecules induce different signaling responses that reflect microbial lifestyle and the subsequent outcome of the interaction.

## Introduction

Throughout their life cycle, plants are exposed to environmental fluctuations that affect their development and functioning. They also have to cope with a range of microbes (e.g., bacteria, fungi, oomycetes) that can be pathogenic or beneficial to plant development. In both pathogenic and mutualistic interactions, plants perceive microbial signals or microbe-associated molecular patterns [carbohydrates, lipids, (glyco)peptides, (glyco)proteins] and develop strategies based on a molecular dialog that involves several specific pattern recognition receptors (Jones and Dangl, 2006; Boller and Felix, 2009). This perception is transduced into signaling events that lead to appropriate responses aimed at controlling the outcome of plant–microbe interactions (PMIs). At first, all microbes (whether pathogenic or beneficial) are initially perceived as invaders and activate similar early signaling pathway events related to plant immunity (Zipfel and Oldroyd, 2017). In the case of beneficial microbes, this response is subsequently attenuated or modified to allow for symbiosis to establish (Zamioudis and Pieterse, 2012). Plants control microbe populations in and around roots through sugar exudation by initiating a molecular cross-talk with heterotrophic microbes. Root sugar exudates serve as a signal for mutualistic interactions, but unfortunately also attract pathogenic microbes (Bais et al., 2006). Sugars represent the main source of energy used by the cellular machinery for growth and development. In plants, sucrose is the main compound transported from photosynthetically active source leaves to sink tissues (young leaves, flowers, fruit, roots, seeds…) *via* the phloem (Van Bel, 2003). Alternatively, sucrose or its by-products can be transferred to microbial sinks. Thus, plant colonization or infection by microbes modifies sugar allocation at the whole plant level and increases carbon sink strength (Biemelt and Sonnewald, 2006; Doidy et al., 2012a). Photoassimilate transport requires specific membrane transporters in both plant and fungal partners. Plant sugar transporters are divided into three families. The sucrose transporter (SUT) (Sauer, 2007; Lemoine, 2000) and the monosaccharide transporter (MST) families (Büttner and Sauer, 2000; Büttner, 2007) both belong to the Major Facilitator Superfamily and mainly co-transport sugars with protons. SUTs are involved in the long-distance transport of sucrose from source leaves to microbial sinks (Doidy et al., 2012a). In colonized root cells, sucrose is exported into the apoplast *via* SUTs and then hydrolyzed into glucose and fructose by plant cell wall or microbial invertases; microbial invertases are only involved in the case of pathogenic interactions (Roitsch and González, 2004). The third sugar transporter family, “Sugars Will Eventually be Exported Transporter” (SWEET), is involved in cellular sugar efflux (Chen et al., 2010). SWEETs play a role in biotrophic exchanges during the development of plant beneficial or pathogenic interactions (Chen et al., 2010, 2015; Yu et al., 2011; Chong et al., 2014; Chandran, 2015; Manck-Götzenberger and Requena, 2016). The capacity of plants to control apoplastic sugar uptake is thus a key determinant for the outcome of these interactions. However, the way microbes manipulate plant carbohydrate transporters at biotrophic interfaces and finally control the outcome of PMIs is still unclear.

To decipher PMI functionality with respect to sugar partitioning regulation, we compared microbial compounds from microbes with opposite trophic strategies. The first of them was cryptogein, a proteinaceous plant defense elicitor secreted by the oomycete *Phytophthora cryptogea.* It induces a hypersensitive response (HR) and systemic acquired resistance (SAR) against various pathogenic microbes in *Nicotiana tabacum* (*N. tabacum*), a non-legume plant (Bonnet et al., 1996). The cryptogein-induced signaling events that lead to gene reprogramming and cell death have been extensively studied (Garcia-Brugger et al., 2006). Within the framework of our study, it is interesting to note that cryptogein blocks glucose uptake in tobacco cells in suspension, with a subsequent decrease in O_2_ consumption (Bourque et al., 2002). This energy deficiency promotes cell death as a defense mechanism. However, the impact of cryptogein on the uptake of other sugars or the expression of genes encoding sugar transporters has never been studied.

Our second microbial compound was a mycorrhizal-factor (myc-factor). Myc-factors include lipochitooligosaccharides (LCOs; Maillet et al., 2011) and short chains of non-acylated chitin chitooligosaccharides (COs; Genre et al., 2013) which are involved in the initial stages of arbuscular mycorrhizal (AM) symbiosis in plants. The nuclear calcium oscillations induced by these molecules in root epidermal cells of legumes and non-legumes (Genre et al., 2013; Sun et al., 2015) can mimic the oscillations induced by AM fungi (AMF) *in planta* (Kosuta et al., 2008; Chabaud et al., 2011; Genre et al., 2013). LCO and short-CO perception by plant cells induces the common symbiotic signaling pathway (CSSP) involved in the establishment of mycorrhizal and rhizobium-legume symbiosis (Gough and Cullimore, 2011; Oldroyd, 2013). LCOs are less effective in promoting Ca^2+^ responses, so we chose COs to induce the early signaling cascade of a beneficial fungal partner. Among short COs, we selected chitotetrasaccharide (CO4) because it is more active in terms of Ca^2+^ spiking in root cells (Genre et al., 2013). We aimed to determine whether the signaling events induced by microbial molecules from organisms with different lifestyles (pathogenic *vs.* mycorrhizal) impacted plant sugar partitioning and in turn sugar exchanges between plants and microbes. Evidence that microbes manipulate the host transport system to increase sugar efflux and that host plants attempt in return to restrict apoplastic sugar availability to halt microbe proliferation is still scarce (Ruan, 2014). In addition, the effect of CO4 as a signaling molecule has never been tested on undifferentiated cells cultured in suspension.

In the present paper, we investigate whether putative differences exist in early responses and sugar exchanges in cells submitted to avirulent pathogen- and symbiont- derived molecules. We report for the first time that undifferentiated cells from a non-legume plant respond to a myc-factor. Hence cell suspensions could represent an efficient tool to decipher the signaling pathways induced by these molecules. We also show that in *N. tabacum* cell suspensions, CO4 and cryptogein trigger the same signaling components [(cytosolic calcium, reactive oxygen species (ROS), mitogen-activated protein kinases (MAPKs)], and expression of sugar transporters yet with specific time courses and intensity levels. These findings suggest that defense and mutualistic responses are finely differentiated in the earliest steps after perception.

## Materials and Methods

### Cell Culture and Preparation

*Nicotiana tabacum* cv. Xanthi cells wild type (wt) and gp15 cells, which express antisense *NtrbohD* under the constitutive *CaMV35S* promoter (Simon-Plas et al., 2002), were cultivated as previously described by Bourque et al. (2011). Briefly, cells were grown in Chandler’s medium (Chandler et al., 1972) on a rotary shaker at 150 rpm at 25°C under continuous light (30–40 μmol.m^-2^ s^-1^). They were sub-cultured to fresh medium [15:100, (v/v)] weekly. For all experiments, 7 day-old cells were diluted to one half in Chandler’s medium and cultured overnight. Before treatments, they were filtrated, washed, and resuspended in I_2_ buffer (2 mM MES, 175 mM mannitol, 0.5 mM CaCl_2_, 0.5 mM K_2_SO_4_, pH 5.8) at a rate of 0.15 g fresh weight.mL^-1^ of I_2_ buffer, and equilibrated for 2 h on a rotary shaker under light (150 rpm, 25°C).

*Nicotiana tabacum* cells expressing cytosolic apoaequorin (Manzoor et al., 2012) were sub-cultured following the same procedure as Xanthi wt cells. Cells were collected during their exponential growth phase, washed by filtration in I_10_ buffer (10 mM MES, 175 mM mannitol, 0.5 mM CaCl_2_, 0.5 mM K_2_SO_4_, pH 5.8), and resuspended at a rate of 0.15 g fresh weight.mL^-1^ of I_10_ buffer. Cell equilibration and *in vivo* reconstitution of aequorin were performed by adding 1 μM of coelenterazine (stock solution in ethanol and storage at -20°C) for at least 3 h in the dark (150 rpm at 25°C) before starting the tests.

### Cell Culture Treatments

All basic salts and chemicals were purchased from Sigma–Aldrich (United States), except coelenterazine that was supplied by Calbiochem (Germany).

Cryptogein was purified according to Bonnet et al. (1996) and stored at -20°C in water as a 50 μM stock solution. Tobacco cells were treated with a final concentration of 50 nM cryptogein. Synthetic CO4 was kindly provided by Guillaume Bécard (LRSV, Castanet-Tolosan, France) and Fabienne Maillet (LIPM, Castanet-Tolosan, France). Stock solutions of CO4 were prepared in water at 10^-3^ M and stored at -20°C. Xanthi cells were treated with a final concentration of 100 nM CO4. Diphenyl iodonium (DPI) was prepared in DMSO and added at a final concentration of 5 μM 5 min prior to CO4 treatment. An equal volume of 0.05% (v/v) of DMSO was added to the control cell suspensions.

### Cytosolic Calcium Measurements

After *in vivo* reconstitution of aequorin, luminescence emission was measured using a luminometer (Lumat LB9507, Berthold Technologies, Germany). For this purpose, as described by (Lecourieux et al., 2005; Manzoor et al., 2012), relative luminescence units (RLU) were continuously recorded every second for 60 min. At the end, residual functional aequorin was quantified by adding 300 μL of lysis buffer (10 mM CaCl_2_, 2% Nonidet-P40, 10% ethanol v/v), and the resulting increase of luminescence was monitored until it reached the basal level. Luminescence data were transformed into Ca^2+^ concentrations ([Ca^2+^]) by using Allen’s equation (Allen et al., 1977): [Ca^2+^] = {(L_0_/L_max_)^1/3^ + 118(L_0_/L_max_)^1/3^ - 1}/{7 x 10^6^ - [7 x 10^6^(L_0_/L_max_)^1/3^}, where L_0_ is luminescence intensity (RLU) *per* second, and L_max_ is the total amount of luminescence in 250 μL of cells.

### Quantification of H_2_O_2_ Production

H_2_O_2_ production was determined by chemiluminescence as described previously by Simon-Plas et al. (1997) with a few modifications. After a 2-h equilibration period in I_2_ buffer, the cell luminescence background of the assay was measured. Then, cells were treated with 100 nM CO4 or 50 nM cryptogein, and 250 μL of cell suspensions were collected and transferred into vials in a luminometer and automatically supplemented with 50 μL of a 0.3 mM luminol solution and 300 μL of I_50_ buffer (50 mM MES, 175 mM mannitol, 0.5 mM CaCl_2_, 0.5 mM K_2_SO_4_, pH 6.5) for RLU measurements for 60 min. At the end, a calibration curve with known amounts of H_2_O_2_ was drawn: the luminescence of untreated cells was recorded to convert RLU into H_2_O_2_. Results were expressed in nanomoles of H_2_O_2_ equivalents *per* gram of cells.

### Protein Extraction

Five mL of cell suspension were sampled 0, 5, 10, 30, and 60 min after cryptogein or CO4 treatment. Cells were harvested by filtration on GF/A fiberglass filters (Whatman International Ltd., United Kingdom), frozen in liquid nitrogen and ground in a mortar. Then 250 mg of cell powder were supplemented with 400 μL of protein extraction buffer (50 mM HEPES pH 7.5; 5 mM EDTA; 5 mM EGTA; 50 mM β-glycerophosphate; 1 mM Na_3_VO_4_; 2 mM DTT; 10 mM NaF and 1 mM PMSF). The extracts were incubated on ice for 15 min and centrifuged at 23,000 × *g* for 20 min at 4°C. Supernatants were stored at -80°C or supplemented with Laemmli buffer (Laemmli, 1970). Protein concentrations were quantified according to the method described by Bradford (1976) using bovine serum albumin (BSA) as a reference for protein concentrations.

### In-Gel Kinase Activity Assays

Ca^2+^-dependent or independent protein kinase activity was analyzed by in-gel kinase activity assays as previously described by Kameshita and Fujisawa (1989), with a few modifications. Twenty μg of protein extracts were analyzed by electrophoresis on 10% sodium dodecyl sulfate–polyacrylamide gel electrophoresis (SDS-PAGE) embedded with two different PK substrates: 0.14 mg.mL^-1^ histone IIIS (HIIIS) or 0.25 mg.mL^-1^ myelin basic protein (MBP). After electrophoresis, SDS was removed by performing two 30-min washes with buffer A (50 mM Tris–HCl pH 8.0; 20% isopropanol v/v), and then two more 30-min washes with buffer B (50 mM Tris–HCl pH 8.0; 5 mM β-mercaptoethanol). Then, the proteins contained in the gel were denatured by two 30-min washing steps in buffer C (50 mM Tris-HCl pH 8.0; 6 M guanidine; 5 mM β-mercaptoethanol) before being renatured in five successive washes, including one at 4°C with buffer D (50 mM Tris-HCl pH 8.0; 0.04% Tween 40 v/v; 5 mM β-mercaptoethanol) for 16 h. The gels were equilibrated for 1 h at room temperature with phosphorylation buffer E (40 mM HEPES pH 7.5; 20 mM MgCl_2_; 2 mM DTT) with 0.5 mM Ca^2+^ or in the absence of Ca^2+^ (0.1 mM EGTA) followed by 1 h in buffer E supplemented with 15 μM ATP and 15 μCi [γ-^32^P]-ATP (PerkinElmer, United States). The phosphorylation reaction was stopped by different gel washes with buffer F (5% TCA w/v; 1% potassium pyrophosphate w/v). Gels were dried 1 h at 80°C (GelDryer, Bio-Rad, United States), and PK activity was revealed by exposure to a PhosphorImager screen (Molecular Dynamics Inc, United States). Autoradiography (Amersham Hyperfilm ECL, United Kingdom) was also carried out.

### Immunoblot Analysis

Ten μg of each protein extract were subjected to 10% SDS-PAGE and transferred to a nitrocellulose membrane (HybondC, Amersham, United Kingdom) by semi-dry electroblotting transfer (Trans-Blot SD, Bio-Rad, United States) for 40 min at 15V, using transfer solution [48 mM Tris; 39 mM glycine; 0.0375% SDS (m/v), 20% methanol (v/v)]. A membrane-blocking step was performed for 1 h in TBS-T buffer [10 mM Tris–HCl pH 7.5; 150 mM NaCl; 0.05% Tween 20 (v/v)] supplemented with 2% BSA (m/v) on a rotary shaker before being incubated 1 h at room temperature, with primary polyclonal phospho-p44/42 MAPK (Erk1/2, Thr202/Tyr204) antibody (1/5000, Cell Signaling Technology, United States) or primary polyclonal ERK1/2 (total) antibody (1/1000, ab196883 Abcam plc., United Kingdom). Then, three washes were performed with TBS-T for 10 min, and the membranes were incubated 1 h at room temperature with an appropriate horseradish peroxidase (HRP)-conjugated secondary antibody (1/10000, Bio-Rad, United States). Immunoreactive proteins were revealed by electro-chemiluminescence (ECL, LumiGLO, Cell Signaling Technology, United States).

### Uptake of Radiolabeled Sugars by Tobacco Cells

Radiolabeled sugar uptake was monitored as previously described by Bourque et al. (2002). Sugar uptake was individually measured after the addition of 2 mM of ^14^C-D-glucose, ^14^C-D-sucrose or ^14^C-D-fructose (0.055 MBq.g^-1^ fresh weight (FW) cells, PerkinElmer, United States) 5 min before CO4 or cryptogein treatment. After various processing times (0, 15, 30, 60 min), duplicates of 2 mL of cell suspensions were taken and filtered *via* a vacuum pump on GF/A fiberglass filters (Whatman International Ltd., United Kingdom). The cells were washed twice: 1 min with 10 mL of I_2_ buffer, and then 20 s with 5 mL of I_2_ buffer. Cell pellets were harvested, weighed, and placed at 80°C for 16 h. Dry weights were determined, and dried cells were mixed with 10 mL of scintillation liquid (Ultima Gold Ready-Safe Cocktail, PerkinElmer, United States) in scintillation vials to evaluate the radioactivity of each sample using a liquid scintillation counter (Beckman Coulter LS6500 Liquid Scintillation Counter, United States). Sugar uptake measurements were expressed in μmol of glucose/sucrose/fructose *per* gram of cell FW as a function of time.

### Cell Death Measurements

Cell death was determined by simultaneously staining cells with fluorescein diacetate (FDA) and propidium iodide (PI) dyes, as described previously (Jones and Senft, 1985). Xanthi cells were incubated at room temperature with 20 μg.mL^-1^ FDA and 1 μg.mL^-1^ PI for 5 min. The ratio of dead (red-stained) to viable (green-stained) cells was measured with an epifluorescence microscope (Photomicroscope Axiophot, Zeiss, Germany) using rhodamine (BP546 ± 12/FT580/LP590 nm) and fluorescein (BP450-490/FT510/LP520 nm) filters, respectively. The experiment was repeated three times with at least 500 cells counted for each assay.

### *In Silico* Analysis

Putative *NtSUTs* and *NtSWEETs* were screened from the Solanaceae database^1^ using BLAST algorithms (BLASTN, BLASTP and BLASTX^2^) with previously published plant *SUT* and *SWEET* sequences as subjects. Microarray data from tobacco cells generated in our laboratory (Agilent 44K chip containing 43,759 probes of tobacco cDNA; unpublished results, K. Bouhidel) led to the identification of 2 putative NtSWEETs, named NtSWEET2A and NtSWEET2B. The accession numbers of the isolated sequences are the following: *NtSUT1-1* (MF140390), *NtSUT1-2* (AF149981.1, published as *NtSUT3*, Lemoine et al., 1999), partial *NtSUT2* (MF140391), *NtSUT4* (AB539539.1, Okubo-Kurihara et al., 2011), *NtSWEET2A* (identified as *N. tabacum* bidirectional sugar transporter SWEET2a-like in the original tobacco genome annotation XM_016629722.1), and *NtSWEET2B* (identified as bidirectional sugar transporter SWEET2-like in the original tobacco genome annotation XM_016634622.1).

### RNA Extraction and RT-PCR Analyses

Five mL of cell suspension were collected 0, 30 min, 2 and 4 h after cryptogein or CO4 treatment. The biological material was harvested, frozen and ground in the same way as for protein extraction. Total RNAs were extracted and purified using RNeasy Plant Mini Kit (Qiagen, Germany) following the manufacturer’s instructions. RNA solutions were treated with DNase (Ambion, United States) for 30 min at 37°C, following the manufacturer’s instructions. Purity and concentrations of RNA fractions were estimated using Nanodrop1000 (ThermoFisher Scientific, United States). Reverse transcription was performed on 1 μg of RNA using the ImpromII^TM^ Reverse Transcriptase kit (Promega, United States), as described by the manufacturer.

Quantitative PCR was carried out on reverse-transcribed RNAs from at least three independent biological replicates and two technical replicates. Quantitative PCR reactions were performed using a ViiA^TM^ 7 Real-Time PCR System (Applied Biosystems, United States) apparatus and ViiA^TM^ 7 v1.2 software, using GoTaq^®^ qPCR Master Mix (Promega, United States). Relative expression levels were normalized against the average of the two housekeeping genes *NtL25* and *NtEF1*α (Schmidt and Delaney, 2010) and against untreated cells for each time-point measurement using the 2^-ΔΔCt^ method (Livak and Schmittgen, 2001). The qPCR primers displayed high amplification efficiency (85–100%); they are listed in Supplementary Table 3.

### Statistical Analyses

Cell death data were statistically analyzed for differences between treatments using R software (a language and environment for statistical computing, R Foundation for Statistical Computing, Vienna, Austria, T RCore - Online^3^) using Student’s *T*-test. Differences were considered as significant with a *p*-value < 0,001. Gene expression data were analyzed for differences between treatments by ANOVA followed by Tukey’s range test. Different letters indicate distinct statistical groups with a *p*-value < 0.05.

## Results

### CO4 Induces a Rapid and Transient Cytosolic [Ca^2+^] Elevation in Tobacco Cells

**FIGURE 1:**
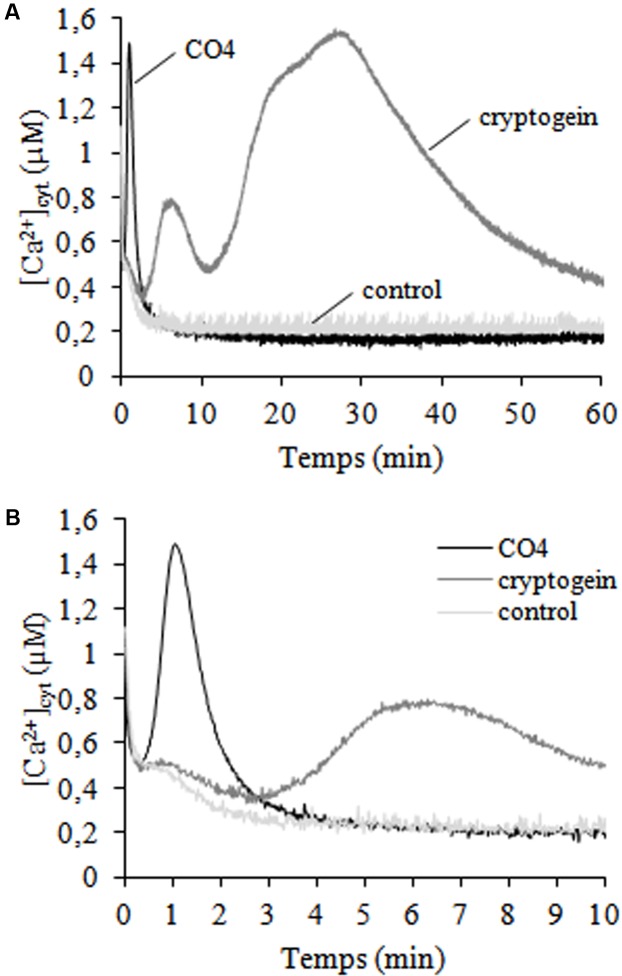
Time courses of cytosolic free calcium concentrations ([Ca^2+^]_cyt_) induced by CO4 and cryptogein. Control, 100-nM CO4- and 50-nM cryptogein- treated cells. **(A)** [Ca^2+^]_cyt_ was measured for 60 min in an *N. tabacum* cv. Xanthi cell line expressing cytosolic aequorin and based on a bioluminescence calibration curve. The curves in graph **(B)** correspond to the first of graph **(A)**. Data correspond to one representative experiment out of four. Mean values ± SEs are given in Supplementary Table 1.

Calcium ions (Ca^2+^) are secondary ubiquitous intracellular messengers that play key roles in signal transduction in response to biotic and abiotic stresses. We investigated the cytosolic free calcium concentrations ([Ca^2+^]_cyt_) dose-response of *N. tabacum* cv. Xanthi cells expressing cytosolic aequorin (Manzoor et al., 2012) and submitted to CO4 treatment (Supplementary Figure 1). CO4 100 nM induced the highest cytosolic [Ca^2+^] rise, so we chose this concentration for the other experiments. A very sharp, rapid and transient increase in [Ca^2+^]_cyt_ was recorded within the first minute of CO4 treatment (**Figures 1A,B**). Then [Ca^2+^]_cyt_ returned to the same basal level as control cells after 5 min of CO4 treatment. These CO4-induced [Ca^2+^]_cyt_ variations markedly differed from the bi-phasic variations induced by cryptogein (**Figures 1A,B**). After a 3-min lag phase, cryptogein induced a first transient [Ca^2+^]_cyt_ increase that peaked after *ca*. 5 min, followed by a second and sustained elevation after 25 min of treatment. The mean [Ca^2+^] peak values obtained with 100 nM CO4 and 50 nM cryptogein treatments and the corresponding peak times are reported in Supplementary Table 1. This result is in agreement with previous data published by Manzoor et al. (2012). Our findings indicate that undifferentiated tobacco cells perceive signals from CO4 used as an AMF inducer and cryptogein used as a plant defense inducer, and then may transduce specific responses to these signals.

### CO4 Promotes Rapid H_2_O_2_ Production Mediated by Plasma Membrane Oxidase NtrbohD

**FIGURE 2:**
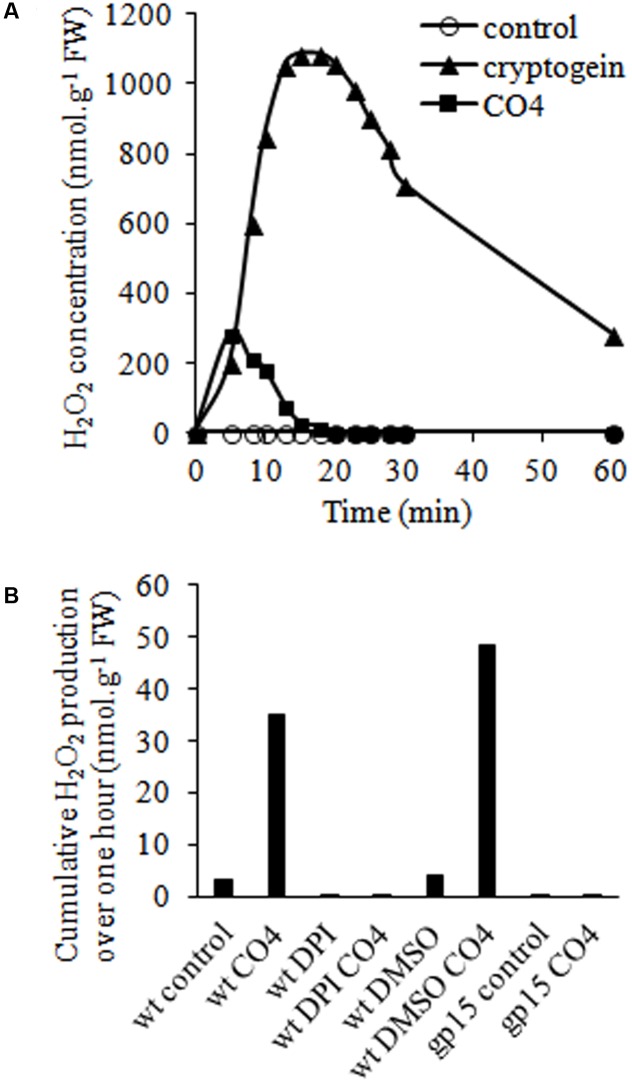
CO4-induced H_2_O_2_ production is mediated by NADPH oxidase (NtrbohD) activity in tobacco cells. **(A)** Time courses of H_2_O_2_ accumulation in wild-type cells treated with 100 nM CO4 or 50 nM cryptogein. H_2_O_2_ concentrations were measured by luminescence. Data correspond to one representative experiment out of five. Mean values ± SEs are given in Supplementary Table 2. **(B)** Impact of NtrbohD activity on CO4-mediated H_2_O_2_ production. Wild type (wt) and *NtrbohD* antisense (gp15) cell lines were pre-incubated or not with 5 μM diphenyl iodonium (DPI) before CO4 treatment. Mean cumulative H_2_0_2_ production was measured for 1 h. Data correspond to one biological replicate out of three.

Reactive oxygen species production is a well-known signaling event triggered by microbial molecules (Fester and Hause, 2005; Torres, 2006), so we investigated H_2_O_2_ production in tobacco cells following CO4 treatment. A rapid and transient increase of the H_2_O_2_ concentration occurred after 5-10 min, with a maximum of 285 nmoles of H_2_O_2_.g^-1^ of FW, followed by a rapid decrease (**Figure 2A**). In the same conditions, cryptogein treatment induced a higher increase of the H_2_O_2_ concentration after around 20 min, with a maximum of 1 μmole of H_2_O_2_.g^-1^ of FW cells, as previously described by Simon-Plas et al. (1997). The mean H_2_O_2_ production peak values obtained with 100 nM CO4 and 50 nM cryptogein treatments and the corresponding peak times are reported in Supplementary Table 2. In order to investigate the origin of H_2_O_2_ produced in response to CO4, we used DPI to inhibit ROS production mediated by flavoenzymes such as NADPH oxidases (Pugin et al., 1997). DPI suppressed H_2_O_2_ production in response to CO4 (**Figure 2B**), suggesting the involvement of NADPH oxidases (also named rbohs for respiratory burst oxidase homologs). To support this hypothesis, we examined CO4-induced H_2_O_2_ production in the gp15 cell line, which is impaired in *NtrbohD* expression by an antisense strategy. This cell line was previously used to confirm that the major enzymatic source of H_2_O_2_ in cryptogein signaling is the NtrbohD isoform (Simon-Plas et al., 2002). Likewise, no H_2_O_2_ production occurred after eliciting gp15 cells with CO4 (**Figure 2B**), confirming that NtrbohD activity is also involved in CO4-induced H_2_O_2_ production in tobacco cells. Similarly to our previous observations about Ca^2+^ (**Figure 1**), H_2_O_2_ production measurements showed that CO4 and cryptogein induced specific profile of oxidative bursts, confirming that myc-factors and plant defense inducers regulate signaling components in specific manners.

### Activation of MAP Kinases by CO4 and Link with Cell Death

Protein phosphorylation/dephosphorylation is a major post-translational modification that plays a crucial role in the regulation of physiological processes by intracellular signal transduction.

**FIGURE 3:**
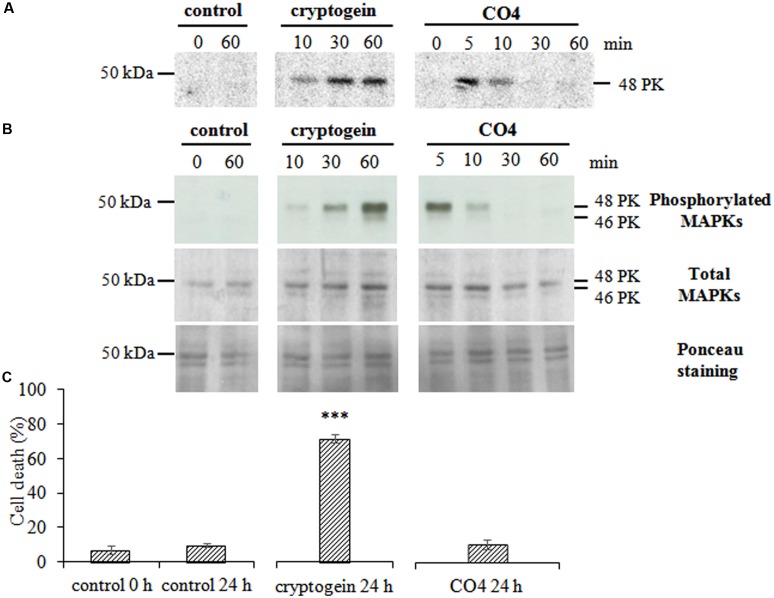
CO4 induces a rapid and transient activation of MAPKs but does not induce cell death. Tobacco cells were left untreated (control) or treated with 50 nM cryptogein or 100 nM CO4, sampled at different time points, and then global protein extracts were prepared. **(A)** Protein kinase (PK) activity levels were analyzed by in-gel kinase assay using MBP as a substrate. The phosphorylation reaction was performed in the absence of Ca^2+^ in the medium. Twenty μg of total proteins were loaded *per* lane. The molecular mass (in kDa) is indicated on the left-hand side of the gels. The entire experiment was repeated independently three times. **(B)** Phosphorylated (active forms) and total MAPKs were immuno-detected using anti-human phospho-p44/42 and total MAPK antibodies, respectively. These data are representative of three independent experiments. **(C)** Cell death was measured by double staining using fluorescein diacetate and propidium iodide in tobacco cells treated or not with cryptogein or CO4 for 24 h. Data are the mean values ± SEs of three biological replicates. Statistical analysis was performed using Student’s *T*-test (*p*^∗∗∗^ < 0.001, *n* = 3).

Using in-gel kinase assays and HIIIS in the presence of 0.5 mM Ca^2+^, we did not detect any Ca^2+^-dependent protein kinase (PK) specifically activated in response to CO4. The activated Ca^2+^-dependent PK patterns were similar for CO4-, cryptogein- and non- treated cells. However, in in-gel kinase assays with MBP in the presence of 0.1 mM EGTA, CO4 activated a Ca^2+^-independent PK with an apparent molecular mass of 48 kDa (**Figure 3A**). Hence, fast and transient PK activity was detected in response to CO4, with a peak after 5 min of treatment. In contrast, sustained activity of a PK with a similar molecular mass was observed from 10 min and up to 60 min in response to cryptogein (**Figure 3A**). This PK shared common characteristics with MAPKs. To confirm this hypothesis, we performed immuno-detection of both activated (i.e., phosphorylated) and total (activated and non-activated) MAPKs by using anti-human phospho-p44/42 MAPK (Erk1/2; Thr202/Tyr204) and total MAPK antibodies, respectively (**Figure 3B**). Two bands corresponding to activated MAPKs with apparent molecular masses of 48 and 46 kDa were detected and confirmed the results obtained in in-gel kinase assays. These PKs could correspond to salicylic-acid-induced protein kinase (SIPK) and wound-induced protein kinase (WIPK), respectively, previously identified in tobacco (Zhang and Klessig, 1997, 1998; Lebrun-Garcia et al., 1998; Zhang et al., 1998, 2000). The use of the antibody raised against total MAPKs revealed bands of similar intensity levels in control, cryptogein-treated and CO4-treated cells, indicating that these MAPKs are constitutively expressed. Therefore, we can conclude that activation of these two MAPKs was due to their phosphorylation in response to CO4 and cryptogein treatments. Several authors hypothesized that MAPK activation was involved in HR-associated cell death (Zhang et al., 2000; Yang et al., 2001; Zhang and Liu, 2001; Menke et al., 2005), so we analyzed the ability of CO4 to induce cell death after 24 h of treatment (**Figure 3C**). CO4, which induced transient MAPK activity, only induced 10% of cell death, i.e., the same percentage as in untreated cells after 24 h. In the same conditions, cell death reached nearly 70% in response to cryptogein, which induced sustained MAPK activity. These results confirm that sustained MAPK activation is necessary to promote cell death, as previously reported by Dahan et al. (2009).

### Effect of Microbial Molecules on Sugar Uptake

**FIGURE 4:**
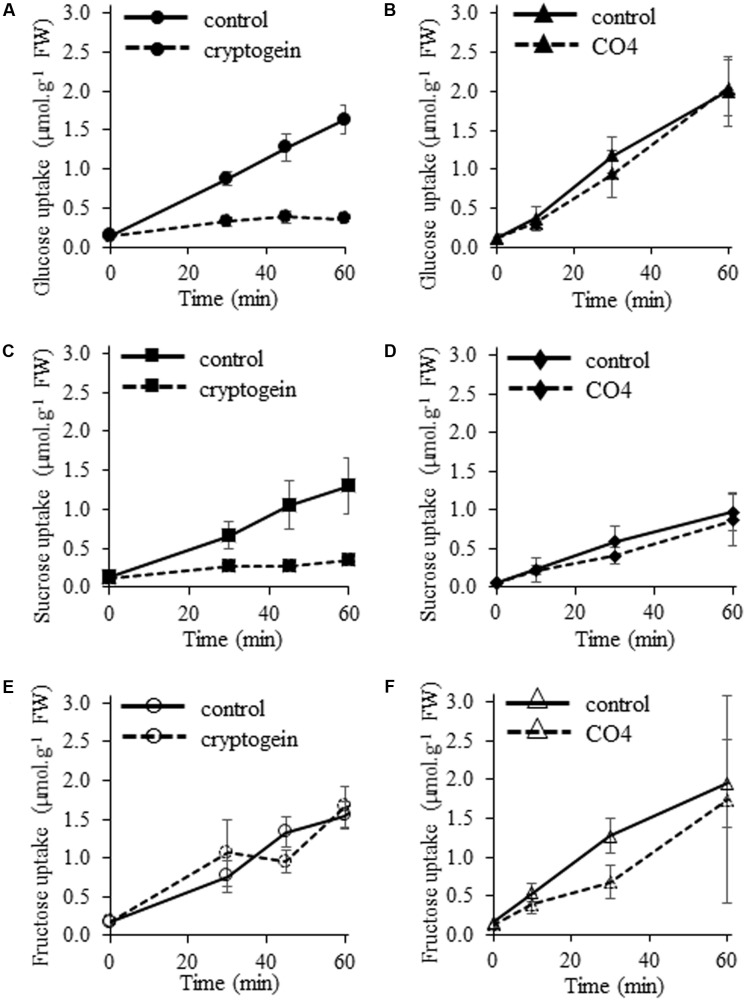
Effects of cryptogein and CO4 on glucose, sucrose, and fructose uptake in tobacco cells. **(A,B)** Time course of ^14^C-glucose uptake. **(C,D)** Time course of ^14^C-sucrose uptake. **(E,F)** Time course of ^14^C-fructose uptake. For all experiments, cells were pre-incubated with 2 mM ^14^C-sugar (0.055 MBq.g^-1^ FW cells), and then 50 nM cryptogein **(A,C,E)** or 100 nM CO4 **(B,D,F)** were added. Controls correspond to the time course of ^14^C-sugar uptake in the absence of cryptogein or CO4. Data represent the means ± SEs of at least three biological replicates.

We analyzed the ability of microbial molecules to regulate cellular uptake of the three main plant soluble sugars, namely sucrose, glucose, and fructose. ^14^C-glucose uptake was totally repressed in cryptogein-treated cells (**Figure 4A**), as previously described by Bourque et al. (2002). In contrast, glucose uptake was not inhibited in response to CO4 (**Figure 4B**). Results were similar for ^14^C-sucrose uptake: CO4 did not modify the sustained sucrose uptake recorded in control cells (**Figure 4D**), but cryptogein strongly inhibited it (**Figure 4C**). These results indicate that sucrose transporter activity and glucose transporter activity are differently regulated in response to cryptogein and CO4. Our observations regarding ^14^C-fructose uptake suggest different modes of regulation. Neither cryptogein treatment (**Figure 4E**) nor CO4 treatment (**Figure 4F**) inhibited fructose uptake as compared to control cells, suggesting that the regulation of specific fructose transporters is not modified in response to microbial signals, or that the regulation of hexose transporters only affects their selectivity. Altogether, our results demonstrate that perception of AMF signals or plant defense inducers leads to adverse strategies in terms of sugar transport at the plant cell plasma membrane, which is related to the subsequent outcome of the interaction.

### CO4 and Cryptogein Differentially Regulate *SUT* and *SWEET* Expression

We focused on the SUT (Zhang and Turgeon, 2009; Kühn and Grof, 2010; Doidy et al., 2012a; Lemoine et al., 2013; Bitterlich et al., 2014; Garcia et al., 2016) and SWEET (Zhang and Turgeon, 2009; Chen et al., 2010, 2012, 2015; Yu et al., 2011; Doidy et al., 2012a; Chong et al., 2014; Chandran, 2015; Manck-Götzenberger and Requena, 2016) transporter families because they are known to play a key role in sugar partitioning *in planta* and in sugar exchanges/competition in PMIs. We first identified all SUT members in the currently available version of the tobacco genome (Sol Genomics Network). Our *in silico* screening led to the identification of 4 putative tobacco *SUTs* (2 full-length and 2 partial sequences) belonging to the three dicotyledonous SUT clades (Supplementary Figure 2). We built a SUT phylogenetic tree based on the amino acid sequence alignment of different plant SUTs (*Arabidopsis thaliana*, *M. truncatula*, *S. tuberosum*, *S. lycopersicum*, and *N. tabacum*) naming NtSUTs according to their phylogenetic position. NtSUT1-1 and NtSUT1-2 belong to the SUT1 clade (type I; Peng et al., 2014), NtSUT2 to the SUT2 clade (type II; Peng et al., 2014), and NtSUT4 to the SUT4 clade (type III; Peng et al., 2014).

In the same database, we also identified 13 putative tobacco SWEETs (full length or at least with a minimum length of 235 amino acids) belonging to the four SWEET clades (Supplementary Figure 3). We renamed the 13 putative sequences according to their closest orthologs with *S. lycopersicum* (Supplementary Figure 3). Among these candidates, our previous data (Agilent 44K, unpublished results, K. Bouhidel) led to the identification of 2 putative NtSWEETs belonging to SWEET clade 1 (NtSWEET2A and NtSWEET2B), that we further investigated.

**FIGURE 5:**
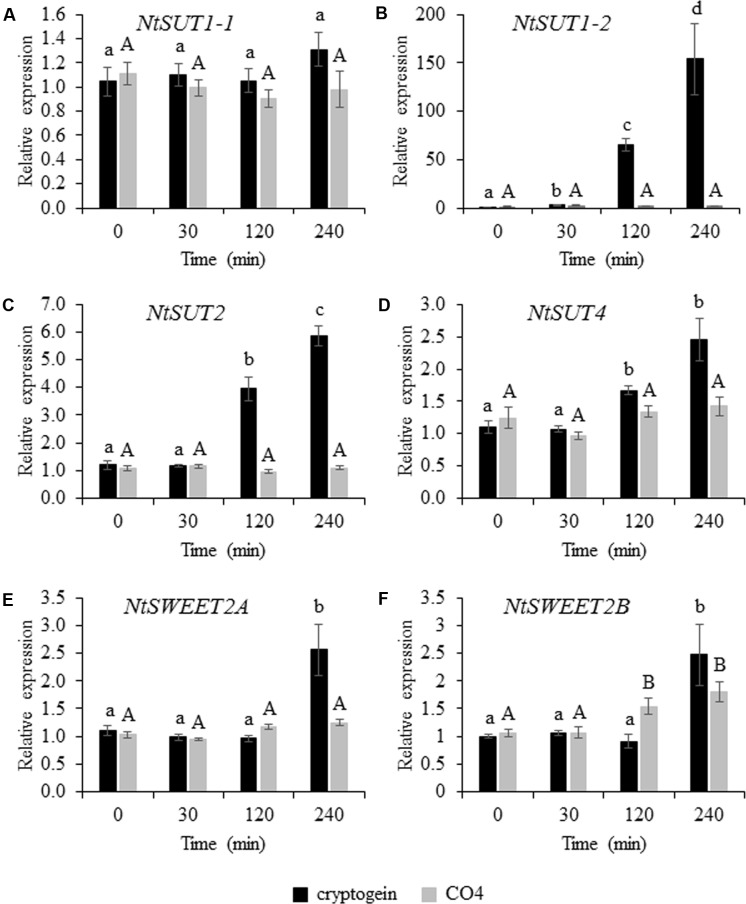
Transcript level of *NtSUTs* and *NtSWEETs* in tobacco cells 0, 30, 120, and 240 min after cryptogein or CO4 treatment. Relative expression of *NtSUT1-1* **(A)**, *NtSUT1-2* **(B)**, *NtSUT2* **(C)** and *NtSUT4* **(D)**, *NtSWEET2A* **(E)** and *NtSWEET2B* **(F)**. Transcript levels were measured in tobacco cells treated with 50 nM cryptogein (black bars) or 100 nM CO4 (gray bars). *NtEF1*α and *NtL25* were used as housekeeping genes, and expression data were further normalized to untreated cells for each time point measurement. These data represent the means ± SEs of three independent experiments. Statistical analysis was carried out using ANOVA followed by Tukey’s test (*p* < 0.05). Capital or lower-case letters correspond to statistical groups following CO4 or cryptogein treatment, respectively.

We analyzed the transcript accumulation of the 6 selected candidates (4 *NtSUT*s and 2 *NtSWEETs*) in tobacco cells by quantitative reverse transcription PCR following 4 h of cryptogein or CO4 treatment. After 4 h of cryptogein treatment, *NtSUT1-1* expression remained unchanged (**Figure 5A**), whereas *NtSUT1-2* transcript levels substantially increased (150-fold change, **Figure 5B**). Transcript accumulation of *NtSUT*2 (**Figure 5C**) and *NtSUT*4 (**Figure 5D**) also significantly increased after 4 h of cryptogein treatment, with 6- and 2.5- fold changes, respectively. In the same conditions, the transcript levels of the two *NtSWEETs* significantly increased 2.5-fold for both genes (**Figures 5E,F**). The changes observed after 4 h of cryptogein treatment occurred after 2 h of treatment for most of the genes we studied (**Figures 5B–D,F**). In contrast, gene expression of all *NtSUT* members remained unchanged in response to CO4 throughout the whole experiment (**Figures 5A–D**). In addition, the 4-h treatment with this symbiotic signal did not modify *NtSWEET2A* transcript levels (**Figure 5E**) and only caused *NtSWEET2B* transcripts to increase slightly but significantly (1.8-fold after 4 h, **Figure 5F**).

## Discussion

We compared the impact of two purified microbial molecules that mimicked pathogenic or mutualistic lifestyles on undifferentiated non-legume cells. We took advantage of this simplified system, which circumvents the hurdles linked to a multi-layered organ, to further unfold the early signaling events of PMI. Our data evidenced that CO4, a myc-factor known to induce the strongest myc responses in root cells (Genre et al., 2013), was recognized by tobacco cells. This recognition resulted in the induction of three signaling events: cytosolic Ca^2+^ production, H_2_O_2_ production, and MAP kinase phosphorylation.

### Early Signaling Triggered by CO4 in Tobacco Cells

Calcium is a key secondary messenger in cell functional processes that transduces early intracellular signaling events in pathogenic and mutualistic PMIs (Vadassery and Oelmüller, 2009). CO4 triggered calcium concentration increase in its very early stages in cultured tobacco cells. Calcium spiking occurs in rhizodermal cells of legume and non-legume plants in contact with AMF or fungal spore/hyphal exudates (Kosuta et al., 2008; Chabaud et al., 2011; Genre et al., 2013; Sun et al., 2015). Calcium initiates the cross-talk between partners, and induces plant (Parniske, 2008) or fungal (Liu et al., 2013) genes, and is thus necessary for symbiosis to establish in the host plant (Oldroyd and Downie, 2006). AMF spore/hyphal exudates contain a mixture of molecules that trigger cytosolic calcium elevations in cultured non-legume (tobacco and soybean) cells (Navazio et al., 2007; Francia et al., 2011). These works are in line with the present study, which more precisely demonstrates that one compound within this mixture, namely CO4, is sufficient to trigger early calcium concentration increase in cells. ROS (H_2_O_2_) production, i.e., the so-called oxidative burst, is a powerful plant defense response against pathogenic microbes (Lamb and Dixon, 1997). But ROS are also generated during the early stages of plant root mycorrhization (Salzer et al., 1999; Fester and Hause, 2005; Kiirika et al., 2012; Belmondo et al., 2015). ROS production is related to induction of carotenoid biosynthesis (Strack et al., 2003), bio-protection of AM plants (Dumas-Gaudot et al., 2000), and lateral root development (Montiel et al., 2013). Our results show that CO4 induced rapid, early H_2_O_2_ production in cultured tobacco cells. They contrast with the work of Navazio et al. (2007) who showed that *G. margarita* culture medium did not induce ROS production in cultured soybean cells. However, a detectable ROS accumulation was recorded in cultured soybean cells treated with homogenates of fungal spore cell walls (Navazio et al., 2007). The authors concluded that the diffusible fungal molecules, which are thought to be released in the medium, did not induce defense reactions in soybean cells. We may wonder whether this undetectable ROS production in soybean cells was due to the absence/low concentration of CO4 (or of other diffusible molecules able to trigger ROS production) or to the presence of ROS antioxidants in *G. margarita* medium that suppressed the oxidative burst. We identified isoform D of Ntrboh as the source of CO4-induced H_2_O_2_ production (resulting from dismutation of O_2_^⋅-^). Rbohs have been described as a major source of ROS during AMF symbiosis (Puppo et al., 2013). In *Phaseolus vulgaris* (Pv), 6 to 9 *Pvrboh* genes were up-regulated during early interaction with the AMF *Rhizophagus irregularis* (Arthikala et al., 2013). *PvrbohB*-RNAi plants developed higher AMF colonization, while *PvrbohB*-overexpressing plants displayed reduced colonization, indicating that PvrbohB negatively controlled the late stage of AMF colonization in cortical cells (Arthikala et al., 2013). Conversely, in *Medicago truncatula* the MtrbohE isoform played a positive role in arbuscule development (Belmondo et al., 2015). Thus, different members of the rboh family could have distinct – sometimes conflicting – functions in AM symbiosis from the very early recognition of diffusible molecules in rhizodermal cells to arbuscule development in cortical cells. We can notice that PvrbohB shares 84.9% of amino acid identity with PvrbohD (Montiel et al., 2012), the NtrbohD homolog. Our data show that CO4 treatment induces NtrbohD-dependent H_2_O_2_ production. This result could be seen in the light of the requirement of PvrbohB for pre-symbiotic early recognition of AMF by rhizodermal *P. vulgaris* cells.

CO4 also induced a transient activation of two MAPKs with apparent molecular masses of 48 and 46 kDa that may correspond to SIPK and WIPK, respectively (Lebrun-Garcia et al., 1998). The MAPK cascade is a key element in the transduction of extracellular stimuli into intracellular response mechanisms (Mishra et al., 2006; Pitzschke et al., 2009). Only few studies have described MAPK involvement in plant mutualistic interactions (Bartsev, 2004; Fernandez-Pascual, 2006; Francia et al., 2011). Induction of MAPK transcripts by *R. irregularis* and *Glomus versiforme* has been measured during pre-contact and appressorium stages on *M. truncatula* roots (Liu, 2003; Weidmann et al., 2004). At the protein level, rapid MAPK activation was triggered in lotus and tobacco cells challenged by *G. margarita* GSE exudates (Francia et al., 2011).

### CO4 and Cryptogein Promote Similar Signaling Events, but with Different Signatures

Our results indicate that the early, subtle, transient induction of [Ca^2+^]_cyt_ production, H_2_O_2_ production and MAPK activation in CO4-challenged tobacco cells was not sufficient to promote tobacco cell death as cryptogein treatment did. The time course and amplitude of these signaling components are decoded by plant cells for them to adapt to different scenarios (Dodd et al., 2010). Interestingly, CO4 and cryptogein induced specific [Ca^2+^]_cyt_ increases that differed in time courses as well as in peak intensities in tobacco cells. CO4 induced a sharp and transient [Ca^2+^]_cyt_ peak, whereas cryptogein induced a longer and bi-phasic [Ca^2+^]_cyt_ elevation, as described by Manzoor et al. (2012). Various external biotic stimuli can quickly trigger specific and distinct spatio-temporal patterns of changes in cytosolic Ca^2+^ concentration, i.e., the so-called “Ca^2+^ signatures” leading to specific signaling pathways (McAinsh and Pittman, 2009). Our results are in agreement with Navazio et al. (2007) and Francia et al. (2011), who showed that AMF *G. margarita* exudates induced a transient [Ca^2+^]_cyt_ increase with a specific sharpened profile within a few minutes in cultured cells. CO4 also induced rapid and transient H_2_O_2_ production within a few minutes, with a peak after 8 min of treatment. The H_2_O_2_ production and [Ca^2+^]_cyt_ production profiles were earlier in CO4-treated cells in cryptogein-challenged cells. Plant rbohs contain EF-hand motifs for calcium binding (Torres and Dangl, 2005), so we expected a similar time course of CO4-triggered calcium and ROS production in link with the “conventional” interconnection between Ca^2+^ and ROS (Mazars et al., 2010). CO4 induced MAPK activation transiently and more rapidly than cryptogein, with a peak after 5 min, while cryptogein induced sustained MAPK activation. This profile is linked with cell death, as suggested by different authors. Constitutive SIPK activation promoted the induction of genes involved in HR cell death (Yang et al., 2001; Zhang and Liu, 2001). Nuclear activation of SIPK resulted in cryptogein-mediated tobacco cell death (Dahan et al., 2009). Moreover, one of the targets of SIPK is WRKY1, a transcription factor involved in HR (Menke et al., 2005). The higher second [Ca^2+^]_cyt_ elevation, which only occurred in cryptogein-treated cells, may be involved in sustained MAPK activation, defense gene expression, and cell death (Lecourieux et al., 2002).

### Impact of Microbial Molecule Origin on Early Sugar Partitioning

Treatment of tobacco cells with CO4 and cryptogein allowed us to characterize the early sugar partitioning events in opposite PMI types. Our tobacco cells perceived the two types of molecules, but the subsequent effects on sugar uptake differed. We showed for the first time that, as previously reported for glucose, sucrose uptake was negatively impacted by cryptogein treatment, whereas fructose uptake was not affected. To discuss our results in the context of competition for apoplastic sugars between plants and microbes, we can hypothesize that (i) microbes inhibit glucose and sucrose re-uptake by mimicking an avirulent pathogen attack so as to divert plant sugars, and/or (ii) plant cells inhibit glucose and sucrose re-uptake to confine pathogen development within the surrounding tissues, and thereby probably promote cell death, as proposed by Bourque et al. (2002). Unlike cryptogein treatment, CO4 treatment did not modify sugar uptake by plant cells. Sugar transfer from the plant to the fungal symbiont is essential for mutualism to develop and lead to reciprocal nutrient exchanges between the two partners. Inhibition of sugar transfer would dramatically impact the outcome of mutualistic symbiosis. Therefore, it is no surprise that plants should maintain sugar flows and maintain sugars available in the apoplast in response to an AMF molecule, so that symbiosis may durably establish. Furthermore, no cell death occurred in CO4-treated cells, in line with the biotrophic obligate lifestyle of AMF. Interestingly, fructose uptake remained unchanged in response to both cryptogein and CO4. Fructose is a by-product of sucrose known as a secondary sugar; it is less competitive than glucose for microbial nutrition. Glucose is mostly used by beneficial microbes, but also diverted by pathogens (Sutton et al., 1999; Hall and Williams, 2000; Voegele et al., 2001; Schüßler et al., 2006; Nehls et al., 2010). The lower selection pressure for fructose uptake may have led to a weaker regulation of fructose re-uptake. This idea is reinforced by the fact that so far only one fructose transporter has been reported in symbiotic or pathogenic fungi, namely BcFRT1 (*Botrytis cinerea* fructose transporter 1; Doehlemann et al., 2005).

### CO4 and Cryptogein Differentially Regulate Sugar Transporters

Plant glucose and sucrose uptake levels can be modified by PMIs such as cryptogein. To offset the ensuing stress, cells may adopt two strategies: (i) favor the synthesis of “other” sugar transporters (located at the plasma membrane and/or tonoplast), or (ii) favor less selective sugar transporters present at the plasma membrane. Additionally, sugar transport proteins may also be regulated at the post-translational level. For instance, phosphorylation of the AtSTP13 sugar transporter in Arabidopsis increased sugar re-uptake by plant cells, and this limited competition with bacterial transporters (Yamada et al., 2016). Our results suggest that at least the first strategy occurs in tobacco cells since cryptogein induced significant up-regulation of 3 *NtSUTs* and 2 *NtSWEETs*, while CO4 only induced a slight but significant increase of *NtSWEET2B* transcripts. Although the expression of 6 transporters was analyzed in our original cell system, it is also interesting to discuss their putative roles at the whole plant level. SUT1 transporters, which belong to the first clade of specific transporters of dicotyledonous plants, are sucrose/proton symporters. Some of them are mainly involved in phloem loading and long-distance transport (Kühn and Grof, 2010). We identified two members of the SUT1 clade, namely *NtSUT1-1* and *NtSUT1-2. NtSUT1-1* was not regulated after short (4 h) treatment with either microbial molecule, whereas *NtSUT1-2* was upregulated after cryptogein treatment, but not after CO4 treatment. Phylogenetic grouping with particular protein orthologs (Supplementary Figure 2) suggests that *NtSUT1-1* is the main sucrose-loading transporter in the phloem, and could be less subject to transcriptional regulation as reported in several higher plants (Zhang and Turgeon, 2009; Doidy et al., 2012b). *NtSUT1-2* is more subject to regulation at both the spatial (e.g., pollen tube; Lemoine et al., 1999) and environmental (e.g., biotic stress as suggested by our results) scales. The main role of SUT2 members remains unknown *in planta*. Regarding our results and the previously proposed negative control of AMF development by SUT2 (Bitterlich et al., 2014), it is tempting to speculate a role for NtSUT2 in the cost/benefit balance of PMIs and its subsequent outcome. CO4 treatment had no effect on SUT2 expression, whereas cryptogein treatment strongly upregulated it. The plasma membrane localization of SUT1 and SUT2 members is clear *in planta*, but localization of SUT4 members is still controversial : they are thought to be localized in the tonoplast, in the plasma membrane, or both (Endler, 2006; Schneider et al., 2012; Chincinska et al., 2013). In cultured tobacco cells, Okubo-Kurihara et al. (2011) suggested that tonoplastic NtSUT4 had a potential role in sucrose export from the vacuole lumen to the cytosol. Interestingly, Schulz et al. (2011) showed that SUT4 proton symporter was not only responsible for sucrose efflux from the vacuole but acted in coordination with TMT1/2, a proton-coupled antiporter (a member of the MST family) that can load glucose and sucrose into the vacuole with a high capacity. Regulation of *NtSUT4* by cryptogein, but not by CO4, suggests an impact on the allocation of sucrose and hexose storage to deplete plant cell sucrose and thereby limit access to carbon sources to fight against a potential pathogen, as previously hypothesized.

Besides SUTs, SWEET transporters represent a novel and widespread type of sugar uniporters in plants. Four plant SWEET subclasses have been identified. Members of subclasses I, II, and IV are mainly monosaccharide transporters, while subclass III proteins are sucrose transporters (Chen et al., 2010, 2012). The physiological roles of SWEETs are multiple, yet they seem specifically involved in diverting nutritional resources from plants in their interactions with both pathogenic and beneficial microbes (Feng and Frommer, 2015). In the present study, the 2 *NtSWEETs* (A and B) belonging to subclass I were upregulated in response to cryptogein. The orthologous protein in Arabidopsis (*AtSWEET2*) encodes a tonoplast protein whose expression is induced in response to infection by the pathogenic oomycete *Pythium irregulare*, and *Atsweet2* mutants are more susceptible to the pathogen (Chen et al., 2015). Thus, AtSWEET2 activity contributes to *Pythium* resistance by preventing sugar loss in the rhizosphere. In our case, only *NtSWEET2B* was upregulated after CO4 treatment. Interestingly, in potato, *SWEET2C* (the closest ortholog of *NtSWEET2B*) is up-regulated during mycorrhization (Manck-Götzenberger and Requena, 2016) and maintains a favorable sucrose gradient in arbuscule-containing cells by mobilizing hexoses in the vacuole. Regulation of *NtSWEET2B* in response to CO4 may initiate the subsequent “sucrose highway.” To sum up, this supports the hypothesis that SWEET2 members may be specifically involved in rerouting nutritional resources from plants in their interactions with both pathogenic and beneficial microbes.

Our study should be placed within a broader contextual framework of PMIs, in which hosts and microbes may develop opposite strategies to acquire carbon molecules at the apoplastic interface (Truernit et al., 1996; Fotopoulos et al., 2003; Azevedo et al., 2005; Chen et al., 2010; Doidy et al., 2012a; Manck-Götzenberger and Requena, 2016; Wittek et al., 2017). The present paper reports for the first time that a myc-factor can be recognized by non-root cells, showing that undifferentiated cells, whatever their origin, are able to perceive the origin of a microbial molecule.

At first, our simplified system was useful to show that in a “mimetic” beneficial symbiotic relationship, signaling features differ from the features of a pathogenic interaction although the signaling cascade involves the same early components as in a pathogenic interaction. This supports the recent hypothesis of a possible overlap between defense and symbiotic responses (Chen et al., 2017).

In a second step, microbes may influence the host transport capacities, whereas plants may respond at later stages by inducing their own transport system most probably through *de novo* synthesis of sugar transporters. The present study contributes to deciphering the complex regulation of sugar exchanges by showing for the first time that two microbial molecules mobilizing the same early signaling events with specific time courses and intensity levels have different impacts on PMI set-up. A way to further unravel the black box of sugar movements across plant membranes during early microbial treatment would be to achieve the functional characterization and the spatio-temporal localization of these key transporters. In the course of evolution, plant–microbe interactions have resulted from long exchanges. While mutualistic (friendly) microbes need to be recognized by plants to be accepted, pathogenic microbes (cheaters) try to mislead the plant so as to be “recognized” as mutualists. Our work illustrates that the “evolutionary battle” is huge and starts with the first signals exchanged by plants and microbes.

## Author Contributions

CP performed the experiments and wrote the paper. SB designed the radiolabeled sugar uptake and in-gel kinase assays. OC, AC, and JF technically supported CP for some experiments. DVT and CP performed *in silico* analyses. DW and NL-C designed the work and wrote the paper. All authors reviewed and approved the final manuscript.

## Conflict of Interest Statement

The authors declare that the research was conducted in the absence of any commercial or financial relationships that could be construed as a potential conflict of interest.

## Footnotes

1 http://solgenomics.net/

2 http://blast.ncbi.nlm.nih.gov/Blast.cgi

3 http://www.R-project.org,2016
