# Supplementary material for: Differential Signaling and Sugar Exchanges in Response to Avirulent Pathogen- and Symbiont-Derived Molecules in Tobacco Cells
Source: Front Microbiol. 2017 Nov 20;8:2228. doi: 10.3389/fmicb.2017.02228 (PMC5701941; doi:10.3389/fmicb.2017.02228)
Supplement: Supplementary file 1 [file Presentation_1.PDF]

Supplementary Material

Differential signaling and sugar exchanges in response to avirulent pathogen- and symbiont- derived molecules in tobacco cells

Carole Pfister*, Stéphane Bourque, Odile Chatagnier, Annick Chiltz, Jérôme Fromentin, Diederik Van Tuinen, Daniel Wipf and Nathalie Leborgne-Castel

*** Correspondence:** Carole Pfister (carolejulie.pfister@gmail.com)

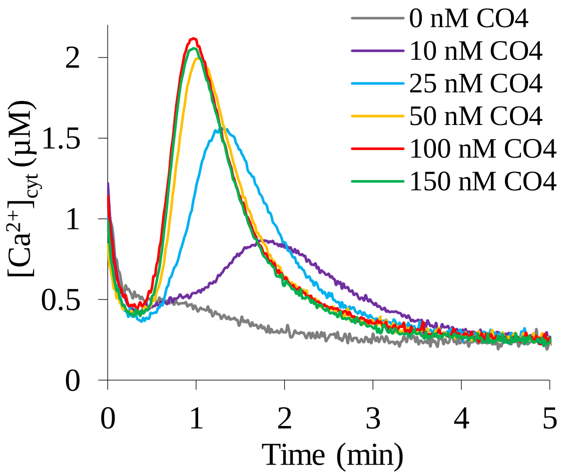

Supplementary Figure 1. Dose-response effect of CO4 on cytosolic free calcium concentrations ($[Ca^{2+}]_{cyt}$) in Xanthi cells expressing cytosolic aequorin.
Cells were treated with 0, 10, 25, 50, 100, and 150 nM CO4. $[Ca^{2+}]_{cyt}$ was measured in an *N. tabacum* cv. Xanthi cell line expressing aequorin in the cytosol and based on a bioluminescence calibration curve.

Supplementary Table 1. Cryptogein- and CO4-induced $[Ca^{2+}]_{cyt}$ variations. Times and amplitudes of the Ca^{2+} peaks (mean values ± SEs, n = 4-5) are given for 50 nM cryptogein (Cry) and 100 nM CO4 concentrations. Peaks 1 and 2 correspond to the first and second $[Ca^{2+}]_{cyt}$ elevations in response to cryptogein, respectively.

	**Peak 1**		**Peak 2**	
	Time (min)	Amplitude (µM)	Time (min)	Amplitude (µM)
Cry-induced $[Ca^{2+}]_{cyt}$ variations	6.90 ± 0.40	0.76 ± 0.09	28.51 ± 2.29	1.69 ± 0.09
CO4-induced $[Ca^{2+}]_{cyt}$ variations	0.99 ± 0.05	2.03 ± 0.36	-	-

Supplementary Table 2. Cryptogein- and CO4-induced H_20_2 production. Time and amplitude of H_20_2 production peaks (mean values ± SEs, n = 5-6) are given for 50 nM cryptogein (Cry) and 100 nM CO4 concentrations.

	**H_20_2 peaks**	
	Time (min)	Amplitude (nmoles of $H_20_2.g^{-1}$ FW)
Cry-induced H_20_2 production	21.00 ± 2.08	719.33 ± 119.18
CO4-induced H_20_2 production	5.60 ± 0.01	236.20 ± 61.56

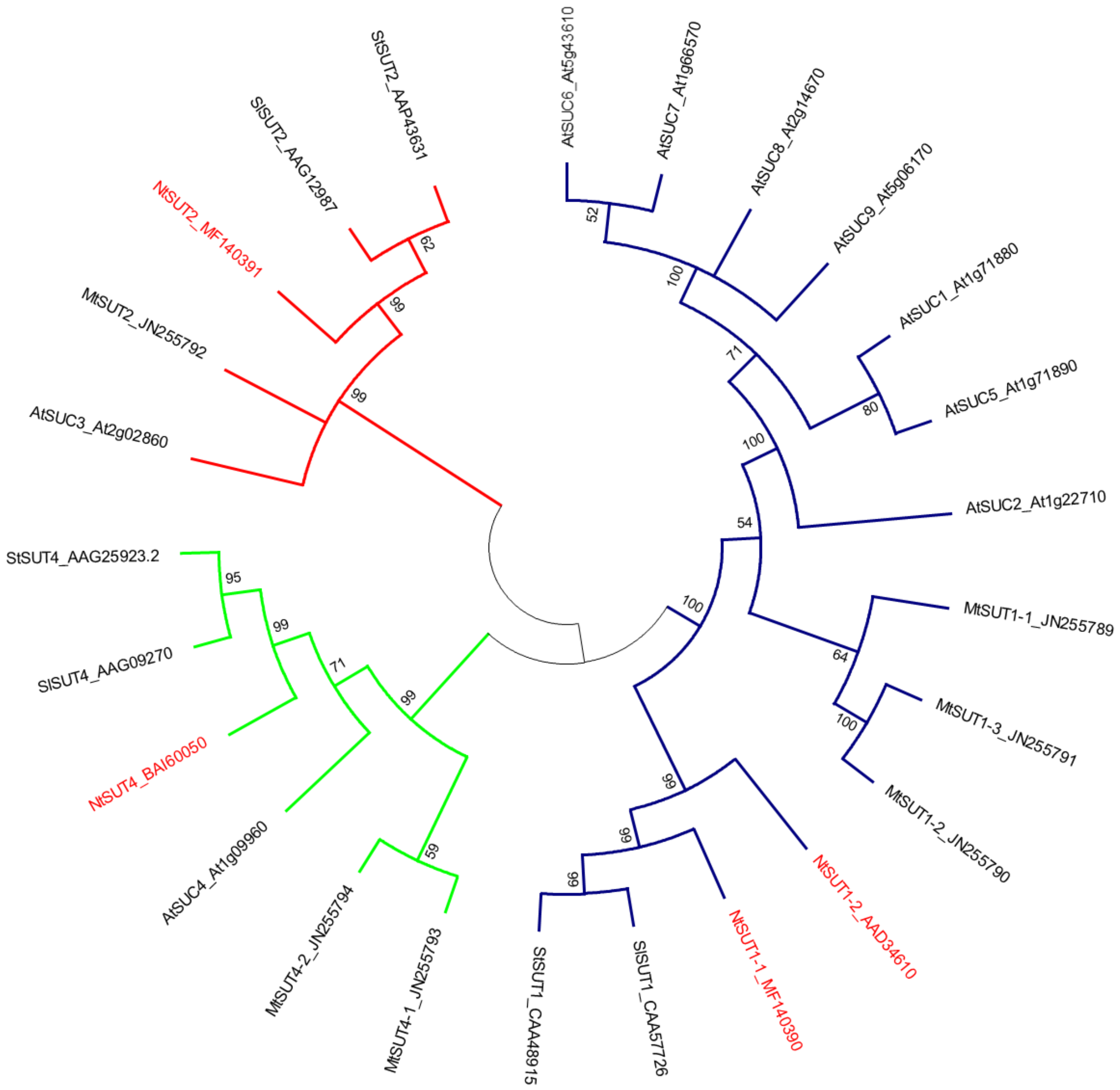

Supplementary Figure 2. Phylogenetic tree of the plant SUT family (*A. thaliana*, *M. truncatula*, *N. tabacum*, *S. tuberosum*, *S. lycopersicum*).

SUT proteins can be divided into 3 distinct clades represented by different colors: SUT1 (blue) (type I; Peng et al., 2014) , SUT2 (red) (type II; Peng et al., 2014), and SUT4 (green) (type III; Peng et al., 2014). The 4 tobacco SUTs are written in red. The phylogenetic tree was constructed using the MEGA7 (Kumar, S., Stecher, G., and Tamura, K. (2016). MEGA7: Molecular Evolutionary Genetics Analysis Version 7.0 for Bigger Datasets. *Mol. Biol. Evol.* 33, 1870–1874. doi: 10.1093/molbev/msw054) program. Amino acid sequences were aligned using the multiple sequence alignment program ClustalW. The tree was constructed using the Maximum Likelihood method corrected by Poisson statistical model and by applying the Neighbor-Joining and BioNJ algorithms with a bootstrap value of 500. The analysis was performed on a total of 25 sequences with 446 phylogenetically informative amino acids. Trees with branches with a robustness value below 50% were removed to obtain strong branches.

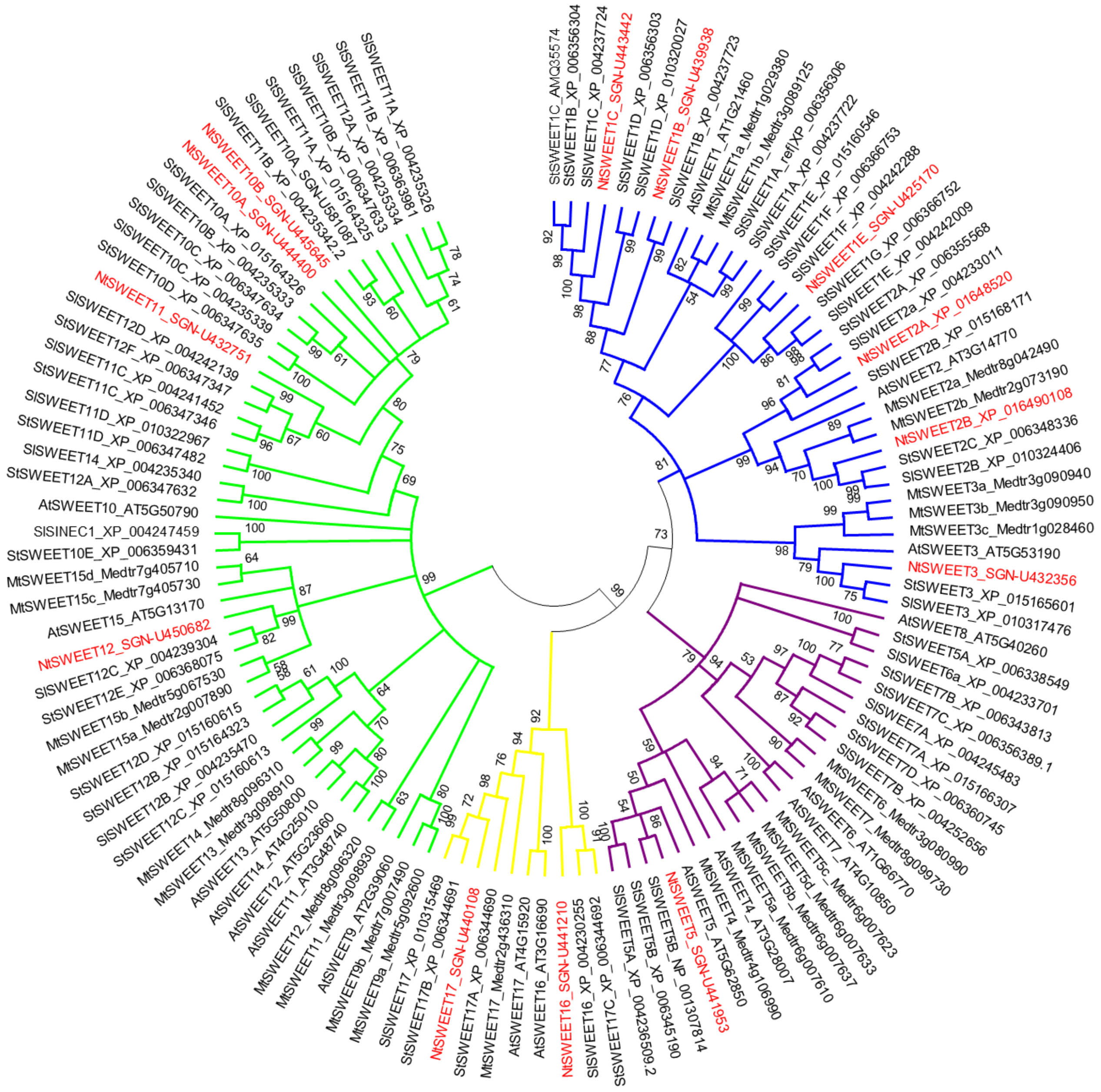

Supplementary Figure 3. Phylogenetic tree of the plant SWEET family (*A. thaliana, M. truncatula, N. tabacum, S. tuberosum, S. lycopersicum*).

SWEET proteins can be divided into 4 distinct clades represented by different colors: clade 1 (blue); clade 2 (magenta); clade 3 (green), and clade 4 (yellow). The 13 tobacco SWEETs are written in red. The phylogenetic tree was constructed using the MEGA7 (Kumar, S., Stecher, G., and Tamura, K. (2016). MEGA7: Molecular Evolutionary Genetics Analysis Version 7.0 for Bigger Datasets. *Mol. Biol. Evol.* 33, 1870–1874. doi: 10.1093/molbev/msw054) program. Amino acid sequences were aligned using the multiple sequence alignment program ClustalW. The tree was constructed using the Maximum Likelihood method corrected by Poisson statistical model and by applying the Neighbor-Joining and BioNJ algorithms with a bootstrap value of 500. The analysis was performed on a total of 119 sequences with 188 phylogenetically informative amino acids. Trees with branches with a robustness value below 50% were removed to obtain strong branches.

Supplementary Table 3. Primers used in the present study, related to Figure 5.

**Primer names**	**Forward primers (5'-3')**	**Reverse primers (5'-3')**	**References**
NtSUT1-1	GGTTCCTACTCCCGCCTCTA	TTTTCGCGGACGACGGTTAGG	this work
NtSUT1-2	GTTCCAGTACTGCCACCACG	GCTGAACAATCATTCCAGAAATAGG	this work
NtSUT2	AGGTTGGCACAGATGGTTTC	AGCGATTGGTTTGCTTGAGT	this work
NtSUT4	AGCCTCTAGATCCCAATCATTGCTC	CACCATAAATTTCTCGACCAAACCA	Okubo-Kurihara et al., 2011
NtSWEET2A	AAG ATG CAG CTG GAA TCG CC	CCA TAT ATG TAT GGC AAT CCT GAG	this work
NtSWEET2B	GAT AGG AGC AAT TCT TGG CAT CAT	TTC CCG CAC AAG AAA AAG AAT CAG	this work
NtL25	CCCCTCACCACAGAGTCTGC	AAGGGTGTTGTTGTCCTCAATCTT	Schmidt et al., 2010
NtEF1α	TGAGATGCACCACGAAGCTC	CCAACATTGTCACCAGGAAGTG	Schmidt et al., 2010
